# Inflammatory Prostatitis Plus IBS-D Subtype and Correlation with Immunomodulating Agent Imbalance in Seminal Plasma: Novel Combined Treatment

**DOI:** 10.3390/diseases12100260

**Published:** 2024-10-18

**Authors:** Roberto Castiglione, Gaetano Bertino, Beatrice Ornella Vicari, Agostino Rizzotto, Giuseppe Sidoti, Placido D’Agati, Michele Salemi, Giulia Malaguarnera, Enzo Vicari

**Affiliations:** 1 Department of Clinical and Experimental Medicine, University of Catania, 95123 Catania, Italy; 2 Research Center “The Great Senescence”, University of Catania, 95100 Catania, Italy; 3 Center of Rare Diseases, Policlinico Catania, University of Catania, 95100 Catania, Italy; 4 Simple Departmental Operating Unit, Internal Medicine Ambulatory Andrology & Endocrinology, ARNAS-Garibaldi, 95123 Catania, Italy; 5 Department “GF Ingrassia” Hygiene and Public Health, University of Catania, 95123 Catania, Italy; 6 Oasi Research Institute—IRCCS, 94018 Troina, Italy

**Keywords:** irritable bowel syndrome, inflammatory prostatitis, IBS severity scoring system, rifaximin, DSF probiotics, interleukins, seminal plasma

## Abstract

We recently demonstrated the effectiveness of long-term treatment with rifaximin and the probiotic DSF (De Simone formulation) in improving urogenital and gastrointestinal symptoms in patients with both chronic inflammatory prostatitis (IIIa prostatitis) and diarrhea-predominant irritable bowel syndrome (IBS-D), relative to patients with IBS-D alone. Because the low-grade inflammation of the intestine and prostate may be one of the reasons for co-developing both IIIa prostatitis and IBS-D, we designed the present study to once again evaluate the efficacy of combined rifaximin and DSF treatment in patients affected by IIIa prostatitis plus IBS-D, but we also measured seminal plasma pro-inflammatory (IL-6) and anti-inflammatory (IL-10) cytokines before and after treatment. Methods: We consecutively enrolled 124 patients with IIIa prostatitis and IBS-D (diagnosed using the Rome III criteria). Patients were randomized into two groups: group A (n = 64) was treated with rifaximin (seven days per month for three months) followed by DSF, and group B (n = 60) was treated with a placebo. By the end of the intervention, 68.7% and 62.5% of patients from group A reported improved NIH-CPSI (National Institute of Health’s Chronic Prostatitis Symptom Index) and IBS-SSS (Irritable Bowel Syndrome Severity Scoring System) scores, respectively, compared to only 3.3% and 5% of the placebo group. Group A patients also had significantly lower mean seminal plasma levels of IL-6 (11.3 vs. 32.4 pg/mL) and significantly higher mean levels of IL-10 (7.9 vs. 4.4 pg/mL) relative to baseline, whereas the levels of IL-6 and IL-10 did not change in the placebo group. Conclusions: The combined treatment with rifaximin and DSF appears to represent the optimal approach for addressing a syndrome such as irritable bowel syndrome (IBS-D plus), which frequently co-occurs with prostatitis (IIIa prostatitis). This approach is particularly beneficial in cases where the symptoms are not always clearly delineated, the etiology is multifactorial, and the diagnosis is multilevel.

## 1. Introduction

Chronic prostatitis/chronic pelvic pain syndrome (CP/CPPS) and irritable bowel syndrome (IBS-D) are somatoform and functional disorders with a prevalence estimated at 11–16% [1,2,3] or approximately 11% of the population globally. Quantifying prevalence can be challenging because only 30% of people who experience IBS symptoms will consult a physician for their IBS symptoms [4,5,6]. These two syndromes, which are often associated with stress and depression [7], can coexist in approximately 30% of patients screened by andrologists and gastroenterologists [8,9,10]. Both syndromes are characterized by a multifactorial pathogenesis, and each condition is defined based on its clinical presentation rather than by clear diagnostic markers or findings. The diagnosis of IBS requires the administration of the Rome III questionnaire [11], while CP/CPSS is identified through the National Institute of Health’s Chronic Prostatitis Symptom Index (NIH-CPSI) [12,13].

In addition to intestinal implications, dysbiosis can also give rise to other significant complications as a consequence of an altered intestinal permeability, which in turn facilitates the migration of bacterial species towards the urogenital tracts. A significant bacterial presence is observed, which breaches the intestinal barrier, which is typically highly selective, in instances of alterations to the intestinal microbiota (dysbiosis). This can result from acute and chronic stress, gastroenteritis, and irritable bowel syndrome, particularly in the diarrheal variant. The etiology of prostatitis in men is the anatomical continuity between the prostate and the intestine, which is very short. Infection and inflammation of the prostate, secondary to intestinal dysbiosis, have irreparable consequences for the quality of the seminal fluid, leukocytosis, and an inflammatory cytokine mediation that can be easily measured for many years (such as IL-6, IL-8, and TNF-α) [8,14]. Observational epidemiological studies have reported an association between inflammatory bowel disease (IBD) and the persistent and chronic inflammation of the prostate, which has been observed to evolve into metaplasia, dysplasia, and cancer. However, a causal link between these two conditions remains to be established and is not included in this study [14,15,16,17].

We recently demonstrated the efficacy of a long-term treatment with probiotics (e.g., DSF) and antibiotics (e.g., rifaximin) in patients with CP/CPPS plus IBS with diarrhea (IBS-D) compared to patients with IBS-D alone. Specifically, the probiotic/antibiotic treatment we tested resulted in a greater response rate of gastrointestinal and urinary symptoms [14,18], as assessed with the IBS Severity Scoring System (IBS-SSS) and the NIH-CPSI. In this case, improvement was defined as a ≥50-point reduction in the IBS-SSS score [19] and a ≥6-point reduction in the total NIH-CPSI score [20]. We found that, after three months of the treatment, 77.7% of patients with NIH-IIIa prostatitis and IBS-D demonstrated a significant (≥50-point) improvement in their IBS-SSS responder rate, and 71.1% of this same patient group demonstrated a ≥6-point improvement in their total NIH-CPSI score [14].

The results of our preliminary study suggested that patients with both CP/CPPS and IBS-D could have a similar underlying pathophysiology, but because the diseases differed in severity, we were unable to draw any clear explanations for the treatment’s mechanism of action. Although the causes of IBS-D are not fully understood, previous research has suggested that IBS-D patients have low-grade intestinal inflammation [21]. Cytokines, which are important modulators of immune responses and inflammatory reactions, can play a central role in intestinal inflammation [22]. Some studies have detected a possible imbalance of circulating cytokines in IBS patients, marked by elevated serum inflammatory cytokines (such as IL-6, IL-8, and TNF-α) and decreased serum anti-inflammatory cytokines (such as IL-10) relative to healthy controls [23,24,25]. The severity of intestinal inflammation has also been associated with the severity of IBS symptoms [26].

Thus, the primary goal of this study was to assess the seminal plasma concentration of the pro-inflammatory cytokine, interleukin 6 (IL-6) and the anti-inflammatory cytokine, interleukin 10 (IL-10) before and after treatment with rifaximin and DSF. The secondary goal was to compare symptom profiles between IIIa prostatitis patients with IBS-D who received the treatment and those that received a placebo. Symptom profiles were measured as the proportion of patients who demonstrated improvement as measured using validated questionnaires such as the IBS-SSS and NIH-CPSI.

## 2. Materials and Methods

**Figure 1:**
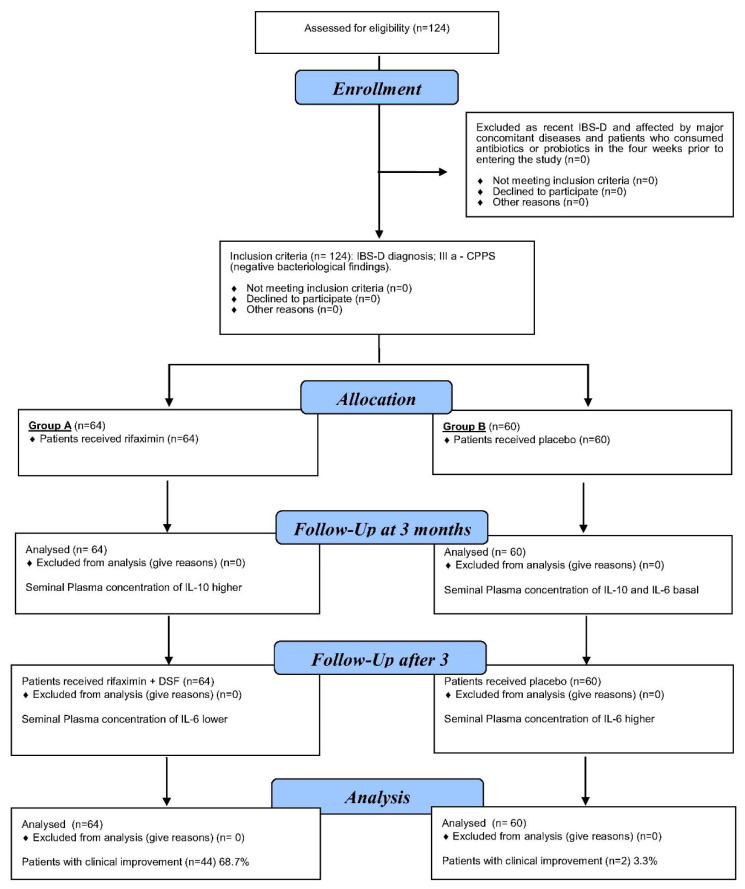
Patients with chronic prostatitis (type IIIa) plus IBS-D, assessed before the treatment and 3 months after treatment with (R + DSF) (group A) or placebo (group B).

We enrolled 124 male outpatients (median age: 56 years; range: 50–68 years) with a confirmed diagnosis of inflammatory chronic pelvic pain syndrome (IIIa prostatitis) and the diarrhea-predominant IBS subtype (IBS-D) (Rome III criteria). Patients were enrolled between January 2016 and February 2019 and were consecutively recruited from the Geriatry Unit Clinic, Post-Graduate School of Geriatry, AOE Cannizzaro, Catania, and Andrology and Endocrinology Unit Clinic, Policlinic University of Catania (Catania, Italy). The diagnoses of IIIa-CPPS and IBS-D were made 24–60 months before the patients were included in this study (Figure 1).

### 2.1. Inclusion Criteria

#### 2.1.1. Diagnostic Rome III Criteria for IBS

IBS-D diagnosis was based on abdominal pain or discomfort for at least three months in the previous six months, with at least two of the following: (i) pain that improved after defecation; (ii) symptoms that were associated with a change in the frequency of defecation; and (iii) symptoms that were associated with a change in the appearance of stool. These symptoms were assessed using a simple “10-point” objective questionnaire based on the Rome III IBS module [27,28]. Patients were diagnosed with IBS-D if they had loose, mushy, or watery stools in the last three months, without any hard or lumpy stools (i.e., question 9 = 0 and question 10 > 0) [27,28].

#### 2.1.2. Diagnostic Symptoms Suggestive of IIIa-CPPS

The presence and severity of IIIa-CPPS symptoms were assessed using the NIH-CPSI questionnaire. In accordance with the EAU guidelines, patients were queried as to whether they had experienced any of the following symptoms for a minimum of three months during the six months preceding this study [29]: pain or discomfort, unrelated to urination, in the pubic or bladder area, perineum, testis, or tip of the penis; ejaculatory pain; pain or burning during urination; incomplete emptying; and frequent urination. A diagnosis of IIIa-CPPS was made when the patient’s NIH-CPSI pain sub-score was greater than 8, indicating a moderate-to-severe level of discomfort [30]. In addition, patients were assigned to the IIIa-CPPS category if any of the following were true: the Meares–Stamey four-glass test [31] produced negative bacteriological findings, the white blood cell (WBC) count in the expressed prostate secretion (EPS) was at least 10 per high-power field (HPF), or the WBC count in the VB3 was at least 5 per HPF [32].

### 2.2. Exclusion Criteria

Exclusion criteria were as follows: (1) a history of chronic bacterial prostatitis (NIH type II) with a positive bacteriological finding on spermculture or on the Meares–Stamey four-glass test [31]; (2) evidence of IIIb-CPSS, defined as an EPS WBC count less than 10 per HPF or a VB3 (Voided Bladder 3) WBC count less than 5 per HPF [32]; (3) obesity (defined as a BMI ≥ 30 kg/m^2^); (4) a history of gastrointestinal bleeding or duodenal or gastric ulcers; (5) patients with chronic or acute illness that could affect this study; (6) patients taking drugs that could affect this study (including anti-inflammatory drugs, PPIs, antidepressants, anti-diarrheal drugs, prokinetics, and antispasmodic agents); (7) patients affected by major concomitant diseases; (8) patients with urinary tract abnormalities or other urological diseases; (9) patients with residual urine volume > 50 mL resulting from bladder outlet obstruction; (10) patients without evident depressive symptoms; or (11) patients who had consumed antibiotics or probiotics within the four-week period preceding their enrolment in this study.

#### 2.2.1. Study Design and Treatment Plan

At their first clinic visit (V0), all 124 patients were randomized into two groups using permuted block randomization with a 1:1 allocation ratio and a block size of four. Patients were assigned random numbers in the order in which they were included and then received the prescription for their respective study group.

Group A (n = 64) was prescribed the non-absorbable antibiotic rifaximin (200 mg tablets; Normix^®^, Alpha Wassermann, Alanno, Italy) and instructed to take two tablets twice a day, seven days per month, for three months. The three-month antibiotic regimen was followed by treatment with the probiotic combination DSF (Visbiome^TM^, ExeGi Pharma, Rockville, MD, USA: 450 × 10^9^ CFU/day). Probiotic powder was taken once daily.

Group B was treated with a placebo. To match the treatment provided to group A, patients in group B were prescribed a placebo tablet (rifaximin-placebo) to be taken seven days each month for three months. The placebo contained sodium starch glycolate (type A), glycerol distearate, colloidal anhydrous silica, talc, microcrystalline cellulose, hypromellose, titanium dioxide E171, disodium edetate, propylene glycol, and red iron oxide E172. For the second half of the intervention, when group A patients were taking the probiotic supplement, the placebo group received a DSF placebo that consisted of microcrystalline cellulose and magnesium stearate, with gelatin as the encapsulating material.

The study protocol was approved by the internal Institutional Review Board of the University of Catania (24/201517 on 17 December 2015), and informed written consent was obtained from each patient. We collected clinical histories from all patients (in both the treatment group and the placebo group), performed a physical examination, and administered the NIH-CPSI and Rome III questionnaires for prostatitis and IBS, respectively [33,34,35,36].

#### 2.2.2. Analytical Measurements

All analytical measurements were performed using blind-coded samples (i.e., no name or personal identifiers). Measurements were taken at the time of enrollment (V0) and again after three months (V3) of the treatment or placebo. For all measurements, we obtained seminal plasma specimens from each participant after 3–5 days of sexual intercourse. Following a minimum 30 min clotting period, the samples were subjected to centrifugation at 1600× *g* for 15 min. The serum samples were then stored at −80 °C until analysis. The levels of IL-6 and IL-10 in the seminal plasma were quantified in duplicate using ELISA kits (Human IL-6 ELISA and Human IL-10 ELISA, BD Biosciences, Milan, Italy).

#### 2.2.3. Assessment of Symptoms

We assessed patient symptoms of IBS-D and NIH-IIIa prostatitis using specific validated questionnaires that patients completed regularly during the treatment period as well as through urological visits performed at the start of therapy (V0) and again three months later (V3).

IBS-D. To monitor the symptoms and changes associated with IBS-D, both groups (treatment and placebo) were requested to complete the IBS-SSS questionnaire on a weekly basis [36,37]. The questionnaire comprises five items, each presented on a visual analog scale (VAS) ranging from 0 to 100 mm. The items assess the severity of abdominal pain (Question 1), the frequency of abdominal pain (Question 2), the severity of abdominal distension (Question 3), the level of dissatisfaction with bowel habits (Question 4), and the extent to which the condition interferes with quality of life (Question 5). The total score is used to classify subjects as having no symptoms (<75), mild IBS (75–174), moderate IBS (175–300), or severe IBS (>300), with total scores ranging from 0 to 500 mm.

Outcome measures for IBS-D. We considered an IBS-SSS score reduction of at least 50 points to be an improvement [15].

Outcome measures for NIH-IIIa prostatitis. Urogenital symptoms were assessed at V0 and V3 using the NIH-CPSI score. We defined a clinically appreciable improvement in NIH-IIIa prostatitis symptoms as a minimum six-point reduction in the total NIH-CPSI score between V0 and V3 [20]. Other endpoints were expressed as the severity of individual CP/CPPS symptoms (measured as scores on the individual NIH-CPSI subscales) as well as WBC counts on EPS after a prostate massage.

#### 2.2.4. Statistical Analysis

The statistical evaluation was conducted using SPSS 9.0 software. The data were expressed as the median and range, and qualitative data were expressed as percentages. The within-group differences in the NIH-CPSI or IBS-SSS questionnaire scores between V0 and V3 were analyzed using Wilcoxon’s signed-rank test. Mann–Whitney U-tests were used to compare the treatment and placebo groups at each timepoint. For all statistical tests, we considered *p* < 0.05 to be significant.

## 3. Results

The two groups of IIIa prostatitis plus IBS-D patients had similar demographic characteristics and baseline clinical and laboratory results (Table 1). At the pre-treatment visit (V0), we detected elevated seminal plasma inflammatory cytokine (IL-6) and decreased serum anti-inflammatory cytokine (IL-10) levels in both groups. At V3, the mean IL-6 level was significantly lower in the treatment group relative to the placebo group (11.3 pg/mL vs. 34.8 pg/mL, respectively; *p* < 0.01) (Table 2). Moreover, we found a statistically significant increase in IL-10 levels in the treatment group between enrolment (V0) and the follow-up visit (V3) (4.4 pg/mL vs. 7.9 pg/mL, respectively; *p* < 0.01). In contrast, serum IL-6 and IL-10 levels were unchanged at the follow-up visit in the placebo group (Table 2).

We also found that the total NIH-CPSI scores decreased significantly (*p* < 0.05) in IIIa prostatitis patients during the treatment, from a baseline mean score of 21.2 to a score 16.4 at V3. All subscales of the NIH-CPSI assessment (i.e., pain, urination, and quality of life) also improved. Overall, 68.7% of the treatment patients (44 out of 64 patients in group A) demonstrated clinical improvement, defined as a ≥6-point reduction in the total NIH-CPSI score. In contrast, patients from the placebo group did not show any significant improvement in their NIH-CPSI scores, neither the total score nor any subscales, during the treatment (Table 2). The percentage of placebo patients that demonstrated improvement (3.3%; 2 out of 60 patients in group B) was correspondingly significantly lower than that in the treatment group.

With respect to gastrointestinal symptoms, symptom severity scores were similar between group A and group B at the baseline visit (Table 1). However, at V3, the IBS-SSS score decreased significantly in the treatment group (mean 162.5, range 115–338) but remained unchanged in the placebo group (mean 272.5, range 175–408). The significant improvement from baseline IBS-SSS scores in the treatment group was associated with a response rate of 62.5% (i.e., 40 of the 64 patients demonstrated a ≥50-point decline), which was also significantly higher than the response rate in the placebo group (5.0%, or 3 of the 60 patients).

Finally, we found that mean leukocyte counts on EPS after a prostate massage decreased significantly in the treatment group (from 13 to 7, *p* < 0.05) relative to the placebo group.

### Compliance

All patients in both groups completed the treatment as planned, and no significant adverse events were reported by any patient during the intervention. However, 7.8% (5 out of 64) of the subjects in the rifaximin group and 9.4% (6 out of 64) of the subjects in the matched-placebo group reported at least one mild adverse event during the first half of the study period (i.e., antibiotic treatment). For this study, mild adverse events were defined as mild and transient symptoms of the gastrointestinal or respiratory tract that could have a possible connection with this study. During the second half of the study period (i.e., probiotic treatment), only 8 of the 64 subjects (12.5%) in the treatment group and 10 of the 64 subjects in the placebo group (15.6%) reported at least one adverse event.

## 4. Discussion

Comorbidities are frequently observed in patients with IBS. These comorbidities may, like IBD, affect the gastrointestinal tract, including functional chest pain, heartburn, dyspepsia, and/or abdominal pain [38,39,40], but they can also produce extraintestinal symptoms, in which case the clinical presentation can be classified in a phenotyping system based on which of the following six domains are affected: urinary, psychosocial, organ-specific, infection, neurologic/systemic, and tenderness (UPOINT) [41,42,43].

A previous study revealed that 31.2% of patients seeking medical advice for PS or IBS in andrological or gastroenterological settings presented with chronic prostatitis and IBS concurrently. These patients exhibited more severe urinary and gastrointestinal symptoms than those with either chronic prostatitis or IBS alone, which we measured as significantly higher scores in the NIH-CPSI (total score and the pain subscale) and the Rome III questionnaire [8]. These results agree with other reports [9,10].

Despite significant investment in research over the past decade, clinical trials have yet to identify effective therapies for IBS patients. Some therapeutic interventions for IBS-D are designed to target presumed IBS-induced alterations in the gut microbiota. These treatments include rifaximin, medical food, serum-derived bovine immunoglobulin, prebiotics, probiotics, and dietary modification. [44]. Rifaximin has been shown to be an effective option for IBS-D treatment [45]: beyond its direct bactericidal effects, rifaximin dampens host pro-inflammatory responses to bacterial products and has demonstrated antibiotic efficacy against isolates derived from patients with small-intestinal bacterial overgrowth [44]. The effects of another treatment option, probiotics, have been less clear; although probiotic supplementation with different (multi-) species pools [46] had beneficial effects on global IBS symptoms and abdominal pain, studies have been unable to draw definitive conclusions about their efficacy [47].

Conversely, patients diagnosed with CP/CPPS are typically treated with an empirical approach. The most prevalent CP/CPPS treatments employed in clinical practice encompass antimicrobial agents and alpha-adrenergic receptor antagonists [28,30,46] or a combined, multi-modal therapy [48,49,50,51].

The results of our controlled study reported here are consistent with the findings from our previous study using a similar treatment [14]: only the rifaximin- and probiotic-treated group had improved symptoms and reduced mean leukocyte counts on EPS after a prostate massage. Thus, we demonstrated that in patients with both NIH IIIa prostatitis plus IBS-D, the administration of rifaximin followed by DSF over a three-month period is effective at alleviating symptoms. For example, 68.7% of the treatment group registered a ≥6-point reduction in their total NIH-CPSI score compared to only 3.3% of the placebo group. Notably, the percentage of our treatment cohort that demonstrated improvement even exceeds the placebo effect of ~64% reported by long-term studies [20]. In addition, the therapeutic intervention also resulted in a significant improvement in IIIa prostatitis symptoms: 62.5% of the treatment group registered a >50-point decline in IBS-SSS scores compared to only 5% of the placebo group. Although there is no consensus on what exactly constitutes a clinically meaningful improvement as a result of a therapeutic intervention, a 50% improvement in the primary endpoint and a 10–15% improvement in the global outcome measure (relative to a placebo group) have been suggested as clinically significant [46].

The results demonstrated a notable alleviation of both urinary and gastrointestinal symptoms, accompanied by a considerable decrease in leukocyte counts on EPS following a prostate massage. The significantly alleviated oxidative stress that we observed after treatment with rifaximin plus DSF implied that an altered gut microbiome was at least partly associated with the pathogenesis of inflammatory prostatitis plus IBS-D. Our combined clinical findings therefore suggested that the therapeutic approach of rifaximin and probiotics should be incorporated into the treatment plan for patients with these coexisting conditions.

Cytokines are key modulators of immune responses and inflammatory reactions and therefore may be involved in the pathogenesis of IBS [22]. Specifically, the low-grade inflammation of the intestine combined with dysbiosis in the gut microbiota may lead to the development of IBS-D and coexisting inflammatory prostatitis. Some studies have suggested that these comorbidities are marked by an imbalance of circulating cytokines, with IBS patients showing elevated serum inflammatory cytokines (IL-6, IL-8, and TNF-α) and decreased serum anti-inflammatory cytokines (IL-10) relative to healthy controls [23,24,25]. Our results agree with previous data documenting significantly alleviated oxidative stress after probiotic supplementation; in our study, probiotic supplementation significantly reduced the serum concentrations of pro-inflammatory cytokines (such as TNF-a, IL-6, and others) and increased the serum concentrations of anti-inflammatory IL-10 [52].

In agreement with a recent systemic review and meta-analysis, which documented after probiotic supplementation a significant reduction in the serum concentration of pro-inflammatory cytokines (such as TNF-a, IL-6, and others) and anti-inflammatory IL-10 [50], in our present study conducted on a group of patients who complained of IIIa prostatitis plus the IBS-D subtype, we documented, in parallel to an improvement in urogenital and gastrointestinal symptoms, in their seminal plasma, after treatment with rifaximin and subsequent DSF probiotic supplementation, a significant reduction in pro-inflammatory IL-6 and an increase in anti-inflammatory IL-10. Therefore, our data indicate that a combined treatment with rifaximin and probiotic supplementation helps to alleviate symptoms and correct seminal plasma cytokine imbalance.

## Figures and Tables

**Table 1:** Characteristics of patient groups with type IIIa chronic prostatitis plus irritable bowel syndrome (IBS-D), after randomization and before treatment.

	Type IIIa Prostatitis Plus IBS-D		
	Group A	Group B	*p* Values
Patients (n)	64	60	ns
Age (years)	55 (50–68)	54 (48–68)	ns
BMI (kg/m^2^)	24 (21–28)	25 (21–29)	ns
Time since diagnosis (months)	37 (24–66)	34 (24–60)	ns
WBC on EPS after prostate massage	13 (10–18)	12 (10–16)	ns
NIH-CPSI total score	21.2 (15–24)	20 (13–24)	ns
IBS-SSS, mean (range)	325 (90–450)	313 (85–433)	ns
>300 (severe) 175–300 (moderate) 75–175 (mild) <75 (no IBS)	27.7 (n = 18) 56.9 (n = 37) 15.4 (n = 10) 0 (n = 0)	24.0 (n = 18) 58.7 (n = 44) 17.3 (n = 13) 0 (n = 0)	
Seminal plasma cytokine levels (pg/mL), mean (range) IL-6 IL-10	30.6 (8–67) 4.4 (2.1–9.3)	28.7 (8–62) 4.7 (2.7–10.2)	ns ns

Irritable bowel syndrome = IBS; BMI = body mass index. Values are expressed as mean and range (in parentheses); ns = not significant.

**Table 2:** Primary and secondary outcomes in patients with chronic prostatitis (type IIIa) plus IBS-D, assessed before the treatment (V0) and 3 months after (V3) treatment with (R + DSF) (group A) or placebo (group B).

	Type IIIa Prostatitis Plus IBS-D			
	Group A (Treatment Group)		Group B (Placebo)	
Study Timepoint	V0 (n = 64)	V3 (n = 64)	V0 (n = 60)	V3 (n = 60)
Primary outcome measures: seminal plasma cytokine levels (pg/mL)				
Mean IL-6 (range) Mean IL-10 (range)	32.4 (10–74) 4.4 (2.1–9.3)	11.3 (5–20) ^#^ 7.9 (4.9–16) ^#^	31.8 (10.5–68) 4.7 (3.3–8.8)	34.8 (12.8–67) 5.2 (3.5–10)
Secondary outcome measures: symptom severity (responder rate)				
Outcome measures related to IIIa prostatitis				
Prostatitis symptoms (NIH-CPSI score)				
Total score	21.2 (15–24)	16.4 *° (10–21)	20 (13–24)	19.7 (13–23)
Pain subscale	11.9 (8–15)	9.0 *° (6–11)	11.7 (8–16)	10.8 (8–11)
Urinary subscale	4.5 (3–6)	3.3 *° (0–3)	4.2 * (3–6)	4.0 (3–5)
Quality-of-Life subscale	4.8 (3–7)	3.8 *° (2–6)	4.8 (3–8)	4.2 (3–7)
NIH-CPSI responder rate (≥6-point decline) No./total No. (%)	NA	44/64 (68.7)	NA	2/60 (3.3)
WBC on EPS after prostate massage	13 (10–18)	7 *° (5–9)	12 (10–16)	10.0 (8–12)
Outcome measures related to IBS-D				
Mean IBS severity score	298.4 (180–410)	162.5 *° (115–338)	307.5 (195–425)	272.5 (175–408)
IBS responder rate (≥50- point decline) No./total No. (%)	NA	40/64 (62.5)	NA	3/60 (5.0)

Irritable bowel syndrome = IBS. Values are expressed as mean and range (in parentheses); NA = not applicable. ^#^ *p* < 0.01 vs. pre-treatment matched values and matched values of placebo group patients. * *p* < 0.05 vs. pre-treatment matched values. ° *p* < 0.05 vs. matched values of placebo group patients.

## Data Availability

Data are contained within the article.
